# The impacts of pricing and reimbursement policies on access to cell and gene therapies across Europe

**DOI:** 10.1007/s12687-026-00860-4

**Published:** 2026-01-26

**Authors:** Yi Han, Mattia Andreoletti, Timo Minssen, Effy Vayena, Kelly E. Ormond

**Affiliations:** 1 Department of Chemistry and Applied Biosciences, Institute of Pharmaceutical Sciences, ETH Zürich, Zürich, Switzerland; 2 Health Ethics & Policy Lab, Department of Health Sciences and Technology, ETH Zürich, Zürich, Switzerland; 3 Centre for Advanced Studies in Bioscience Innovation Law (CeBIL), University of Copenhagen, Copenhagen S, Denmark

**Keywords:** Healthcare equality, Patient access, Health technology assessment, Managed entry agreements, Gene therapy

## Abstract

The increasing number of expensive cell- and gene-based therapies (CGT) on the market have raised concerns regarding equal patient access, regarding both affordability and timely access. Health Technology Assessments (HTA) and Managed Entry Agreements (MEA) play a pivotal role in pricing and reimbursement (P&R) decisions for expensive drugs in Europe, aiming to ensure patient access while optimizing resource allocation. However, discrepancies in HTA and P&R processes lead to delayed, unequal or lack of access in different countries, exacerbating health inequalities across Europe. Though they facilitate access, MEAs introduce additional delays in pricing and reimbursement discussions, and their lack of transparency increases administrative burden and encourages pricing inequity, negatively affecting healthcare system sustainability. Recent legislative changes under Regulation (EU) 2021/2282 have established the Health Technology Assessment Coordination Group (HTACG), which will oversee mandatory joint clinical assessments in the coming years. This new legislation aims to streamline HTA processes across the EU. To address the ethical imperative of improving access equality for CGT in Europe, we propose further policy reforms including concurrent HTA, early benefit assessment, and incorporation of additional elements of value in HTA evaluations, alongside current initiatives to increase cross-border collaboration.

## Introduction

The surge in costly cell- and gene-based therapies (CGT) in recent years has sparked intense debate, fueling demands for reforms in drug pricing and payment methods in all global markets (Carr and Bradshaw 2016; Hampson et al. 2018). Ensuring access to new expensive therapies is increasingly challenging, especially as more high-cost drugs enter the market. Because list prices for these drugs are often unaffordable to pay out-of-pocket, there must be robust reimbursement frameworks in place to allow timely and equal access, particularly to treatments for severe or urgent indications. However, it is equally critical to balance timely patient access with long-term affordability and sustainability of the healthcare system.

The aim of this article is to move beyond discussions of pricing to highlight access challenges raised by the current price-control mechanisms and pricing and reimbursement (P&R) frameworks in Europe for expensive drugs, specifically concerning access across different countries. Much of the literature around accessibility of gene therapies discusses two key themes. First, it often discusses the concept of a “fair price,” and how that might be determined (Garrison et al. 2019; White 2020; Gonçalves 2022; Risse et al. 2024). While these therapies remain largely inaccessible due to their high prices and the systematic barriers those prices create, the notion of price fairness is separate from the issue of patient access. Second, existing literature often centers on cost-related barriers to access in the US (Hemphill 2012; Cornetta et al. 2018; Balderrama et al. 2020; Tsou et al. 2021) or lower-income countries such as Sub-Saharan Africa and India (Bolous et al. 2022; Cornetta et al. 2022; World Economic Forum 2022). Discussion of patient access in these settings often focuses on listed drug prices and decentralized, private reimbursement structures. Patients with better ability to pay, with private insurance coverage, or living in jurisdictions with policies that have higher reimbursement rates and broader coverage can often access higher standards of care, thus having better health outcomes (Niu et al. 2013; Hanna et al. 2022). Access to expensive drugs in Europe, though influenced by access to private coverage and jurisdictional disparities, is often determined primarily by broader national policies and frameworks (Scholz 2020; Palm et al. 2021). Disparities in patient access to gene therapies arise not only from their high costs, but also from national or regional reimbursement decisions shaped by each country’s healthcare budget and value systems. Priority setting is particularly difficult for rare diseases, for which drugs are usually priced much higher to compensate for the low patient number (Drummond et al. 2007). Decisions on healthcare resource allocation for rare indications are further complicated by uncertainty in assessing cost-benefit and added value; difficulty in collecting long-term clinical data for small patient populations limits the ability to make conclusions about safety and efficacy.

To ensure that healthcare resources are allocated efficiently and fairly, European governments have instituted Health Technology Assessments (HTA) as a gate-keeping mechanism. More recently, Managed Entry Agreements (MEA) have emerged as promising tools to facilitate P&R negotiations. After a new therapeutic product has been approved, HTA are conducted on a national level to systematically evaluate the medical, social, economic, organizational, and ethical impacts of new treatments, which provides rationale for P&R negotiations in each country (European Federation of Pharmaceutical Industries and Associations 2023). The primary goals of HTA are to ensure that the prices of new health technologies accurately reflect their clinical effectiveness, and to optimize resource allocation towards efficacious treatments. The HTA framework helps regulate list prices and allows health systems to maximize healthcare outcomes on a limited budget, enabling the maintenance of publicly funded healthcare systems. MEAs are a policy tool used to manage uncertainty and improve patient access to high-cost therapies. They are contracts between manufacturers and national health authorities or payers used during P&R negotiation that assign conditions to the reimbursement of a new health technology (sometimes referred to as conditional reimbursement mechanisms, risk-sharing agreements, or patient access schemes). When HTA is complicated by uncertain clinical and cost effectiveness, MEAs may facilitate a positive reimbursement decision for drugs that otherwise would have been rejected (Efthymiadou and Kanavos 2022). There has been an increased reliance on MEAs to arrive at reimbursement decisions for costly innovative drugs such as CGT (Morel et al. 2013; Wenzli and Chapman 2019; Ronco et al. 2021).

Given recent approvals and discussions of expensive gene therapies (United States Securities and Exchange Commission 2023a, b), we use costly genetically engineered therapies as a case in point to explore the accessibility considerations arising from HTA and MEA processes, though these arguments may apply broadly to innovative and high-priced therapies (Table 1). We center our discussion of healthcare equality for rare diseases on an international scale, examining disparities between countries within the EU, rather than focusing on individual patients or patient subsets. We identify sources of access inequalities stemming from procedural factors, such as inefficiencies in evaluating and negotiating reimbursement for new health technologies, as well as differences in national value systems that shape P&R decisions. Resulting delays and disparities in access raise ethical concerns about equal distribution of treatment, and may contribute to widening gaps in health outcomes for patients with rare diseases.

**Table 1:** Summary of the facilitators and barriers to CGT access identified in this paper related to the use of HTA and MEAs

Health Technology Assessment (HTA)	Managed Entry Agreements (MEA)
Facilitators for patient access	
● Ensure drug prices reflect value to patients and healthcare systems. ● Limit expenditure on inefficacious drugs and encourage uptake of more clinically- and cost-effective drugs. ● Transparency and accountability of reimbursement decisions.	● Distribute financial risk between payers and manufacturers. ● Allow access to urgent treatments with long-term clinical uncertainty. ● Outcomes-based MEAs encourage collection of real-world data.
Barriers to patient access	
● Delay due to HTA directly reduces patient access. ● Differences in speed of HTA introduces patient access inequalities in different countries across Europe. ● Stringent HTA evidence requirements can be more difficult for CGTs for rare diseases to meet	● The lengthy negotiation period for outcomes-based MEAs further delays patient access. ● Lack of transparency is a barrier to international harmonization and collaboration. ● MEAs may amplify pricing inequalities between countries. ● MEAs have uncertain long-term benefits, and their usefulness cannot be evaluated due to confidentiality contracts.

New legislation under Regulation (EU) 2021/2282 facilitates the establishment of The Coordination Group on Health Technology Assessment (HTACG), consisting of HTA bodies and health authorities from all EU states and 3 EEA observers (European Commission n.d.). This regulation “aims to improve the evidence base for assessing new health technologies (e.g. new medicines and medical devices) and to support European Union (EU) Member States in taking timely and evidence-based decisions on patient access” (European Union 2022). Joint clinical assessments in the EU will become mandatory for all ATMPs and oncology products in 2025, and for orphan drugs in 2028 (European Commission 2021). There is hope that this will help streamline HTA across EU countries, cutting time in country-specific approvals while still allowing countries to make their own value judgements during P&R negotiations. Some of the challenges arising from the HTA-P&R process are expected to be addressed by the new legislation, while others remain unresolved, which we aim to address in this article.

## Principles and purposes of HTA and MEAs

The high costs of some CGT, combined with the complex processes required to bring them to market and the lack of long-term data on their safety and efficacy, presents a significant challenge for European national health authorities when it comes to pricing and reimbursement decisions. Both HTA and MEAs are designed to provide a balanced approach for incorporating new therapeutic products into national healthcare systems. HTA provides an evidence-based framework for evaluating the clinical and economic impact of therapies, while MEAs offer the operational flexibility needed to manage uncertainties and financial risks of providing access to innovative technologies. The HTA evaluation is not only a clinical- and cost-benefit evaluation. It also considers a product’s broader impact on the healthcare system, incorporating economic, legal, ethical, and societal dimensions into healthcare decision-making. Clinical benefit is evaluated using tools like Quality-Adjusted Life Years (QALY). While imperfect, QALYs and other tools increase the likelihood that reimbursement decisions are based on relevant patient-centered outcomes, rather than just clinical endpoints. By grounding pricing and reimbursement decisions in evidence-based assessments, it lessens spending on inefficacious drugs and encourages the uptake of more clinically- and cost-effective drugs, so that healthcare investments deliver value to individual patients and society as a whole. Finally, HTA fosters transparency and accountability in healthcare decision-making by providing publicly available reports to explain the rationale behind reimbursement decisions.

It is often challenging to address the financial and evidence-related uncertainties posed by innovative high-cost therapies. The use of MEAs during P&R negotiations may help mitigate the financial risks for payers while ensuring that treatments can be made available to patients while clinical data is being gathered. For example, some MEA models allow reimbursements to be granted temporarily while additional clinical assessments are conducted (Vreman et al. 2020). Financial-based MEAs aim to reduce total expenditure on a product by implementing cost-control strategies, such as budget caps, price-volume agreements, and confidential discounts. Outcomes-based MEAs tie reimbursement to performance in individual patients; the manufacturer will only receive reimbursement for the product when specified clinical outcomes are achieved. Outcomes-based MEAs can offer healthcare systems more flexibility when reimbursement decisions are difficult, while encouraging the collection of real-world data, which in turn helps refine our understanding of a therapy’s true effectiveness and safety over time.

## Access inequalities introduced by the P&R process: new legislative solutions and remaining gaps

HTA and MEAs aim to balance access to treatments with sustainability considerations, offering a framework to integrate costly therapies like CGTs into national healthcare systems while maintaining financial stability. However, implementation challenges may introduce trade-offs, including delays and inequalities in access to new therapies in different countries. Because new products typically cannot enter the market before completion of the P&R process, the HTA and P&R processes, though essential, introduces the most significant delays to drug availability in the EU compared to the US. In the context of gene therapies for severe, progressive genetic diseases, delaying access to novel therapies can lead to significant health and life-year losses due to the irreversible nature of their indications (Coyle et al. 2020; European Federation of Pharmaceutical Industries and Associations 2023). The gravity of access delays is especially pronounced for progressive genetic diseases with no existing treatments. While these delays reflect a necessary caution to ensure value for money, they can pose an opportunity cost on current patients who face irreversible disease progression and lost therapeutic opportunity.

### Barriers to access arising from HTA processes

The clinical effectiveness evaluation of therapies like CGT during HTA raises concerns about the adequacy of traditional assessment frameworks for innovative technologies. The safety and efficacy of a health technology are accepted as the main informers of risk-benefit assessments. Existing HTA evaluations delineate specific requirements to demonstrate evidence of clinical and cost effectiveness, which are challenging to fulfil for disruptive health technologies such as CGT, complicating pricing and reimbursement decisions. Most gene therapies are orphan drugs and are susceptible to assessment pitfalls common in rare disease treatments (Zhou et al. 2022; Palomo et al. 2023). As such, these clinical trials frequently fail to provide adequate evidence to meet the traditional clinical effectiveness criteria used in HTA, leaving evaluators grappling with a high degree of uncertainty regarding long-term safety and efficacy. Small patient populations, short follow-up periods, and reliance on surrogate endpoints make it difficult to generate statistically robust data or assess long-term outcomes. HTA bodies must make recommendations based on incomplete evidence. In such cases, where an “acceptable” amount of evidence cannot be achieved, healthcare authorities are put in a position of weighing patient protection against current patients’ “right to try” (Andreoletti and Blasimme 2023). This uncertainty complicates cost-effectiveness evaluations, leading to prolonged P&R negotiations. Resulting delays in access can have profound negative implications on clinical outcomes for patients with progressive or life-threatening conditions.

Another concern is that the potential broader clinical and economic benefits offered by gene therapies are not captured by traditional QALY and incremental cost-effectiveness ratio (ICER) calculations commonly used in HTA (Drummond et al. 2019; ten Ham et al. 2020; Angelis et al. 2020; Coyle et al. 2020). Existing cost-benefit measures may not fully capture the societal value of CGTs, so certain benefits considered important by patients or policymakers are likely to fall outside the scope of HTA decision-making. Beyond direct health benefits, gene therapies provide additional values, such as the potential to cure debilitating or fatal diseases, adherence improving factors, increased productivity, reduced caregiver burden, and other benefits which are often not considered during a typical cost-effectiveness analysis (Garrison et al. 2019; Qiu et al. 2022). However, it is worth noting that quantifying the impact and assigning these additional values a monetary value is challenging due to their often abstract and multifactorial nature. Taking additional value elements into account may in fact complicate and extend the HTA process, which would be at odds with the goal of timely patient access.

The final challenge lies not in the different P&R decisions that each health authority makes, but in ensuring that HTA bodies are sufficiently resourced to evaluate complex therapies efficiently and effectively. The EU’s member states have diverse healthcare structures and reimbursement policies, shaped by each nation’s social, cultural, and political values. For this reason, each country has its own HTA body. However, the wide variation in HTA budgets, number of available experts, and varying P&R policies for advanced therapy medicinal products (ATMPs; the regulatory pathway for CGT in the EU) affects the timing and likelihood of market access in each country. Due to limited administrative capabilities or research resources, there are differing levels or delays in access to drugs across different countries. Disparities in HTA speeds lead to unequal and inconsistent access to new treatments across the EU, which are likely to adversely impact healthcare outcomes in countries that are slower to conduct HTA (Akehurst et al. 2017; Leinwand et al. 2019; Uyl-de Groot et al. 2020). The median time to access ATMPs in Western Europe ranges from 9 to 17 months, with clinical implementation sometimes taking an additional 2–3 months (Iglesias-López et al. 2023). Patients in less economically developed countries tend to experience later, more limited access. For example, the mean time to availability of orphan drugs in Poland was 32.6 months during 2017–2020 (Newton et al. 2022). Some countries have delayed access because they must wait for others to publish their HTA reports rather than conducting their own, due to budgetary and research limitations. These reflect structural weaknesses within current HTA processes, which disproportionately affect countries with fewer financial and institutional resources. If HTA systems were more effectively coordinated at the European level, there is potential to reduce, rather than reinforce disparities, ensuring more equitable access to innovative therapies regardless of national capacity to conduct HTA.

Regulation (EU) 2021/2282 seeks to address this issue by mandating joint clinical assessments across the EU, with the HTACG facilitating collaborative HTA evaluations to minimize administrative burden, reduce duplication of work, and promote greater alignment in clinical effectiveness evaluations across member states (European Commission n.d.; Julian et al. 2022). Under this new HTA framework, less resource-rich countries with limited ability to conduct HTA would gain access to centralized clinical assessment resources and expertise. This has the potential to equalise P&R decision-making timelines while still maintaining each country’s authority to make its own P&R decisions. This could significantly alleviate some of the longstanding HTA-related challenges across the EU. However, as it currently stands, the legislation addresses only a portion of the broader P&R-related access equality issues.

### Barriers to access arising from divergent P&R decisions

There are different thresholds of risk tolerance, based not only on scientific evidence, but also on cultural and societal values (European Group on Ethics in Science and New Technologies 2021). Finding the right balance for orphan diseases is often challenging because these patients have no alternative treatment options. These decisions vary from one country to the other, and an “acceptable” amount of evidence will differ depending on the level of risk healthcare authorities are willing to take. On an individual level, access to treatment may differ based on nationality or country of residence. Whether these differences are ethically problematic depends on whether providing access to these treatments is considered an ethical obligation. While countries are entitled to make healthcare resource allocation decisions that align with their value systems, it is also important to recognise that divergent approaches contribute to a fragmented landscape and unequal patient access across the EU.

Different economic policies across EU member states may also contribute to indirect access delays. More stringent reimbursement decisions in certain countries often result in slower market entry or limited availability of new therapies in those countries, as manufacturers tend to prioritise new product launches in countries with less restrictive pricing policies to encourage external referencing of higher list prices (Danzon and Epstein 2012). This leads to countries with lower expected list prices consistently experiencing longer launch delays, both across the EU and globally (Danzon and Epstein 2012; Kanavos et al. 2020). Despite a high level of marketing authorization (MA) agreement between the FDA and EMA, CGT have had comparably limited uptake in Europe due to negative or restricted reimbursement decisions in some countries (Michelsen et al. 2020). Patients in Europe may suffer from complete lack of access to innovative treatments due to rejections, delays, withdrawals, or fewer MA applications. Most withdrawals of ATMPs in the EU occur due to concerns about market viability rather than safety and efficacy (Aguilera-Cobos et al. 2022). Manufacturers may choose to withdraw products they anticipate will be unprofitable after receiving national pricing or reimbursement decisions, resulting in a lack of access across entire countries. A notable recent example is the EU-wide withdrawal of Zynteglo, a gene therapy designed to treat transfusion-dependent β thalassemia (European Medicines Agency 2022). The manufacturer failed to secure a favorable listing price for Zynteglo in Germany and lacked confidence in negotiations with other European countries, ultimately leading to the withdrawal of Zynteglo from all European markets in 2021 (Dunleavy 2021). Having withdrawn manufacturing operations in Europe, the company pre-emptively withdrew their second gene therapy product, Skysona, and are unlikely to seek MA for Lyfgenia (European Medicines Agency 2021; Dunleavy 2021). Manufacturers consider the significant infrastructural costs associated with providing CGT unsustainable due to low profit margins. Smaller companies in particular struggle to afford the high upfront costs required to ensure EU market access without the promise of adequate returns. These instances set a precedent for restrictive pricing of CGT in the EU, potentially dissuading manufacturers from investing in MA and infrastructure for similar products in the future. In the eyes of industry stakeholders, the HTA process in the EU not only imposes a profit cap but also leads to a substantial delay to market access, making the unregulated pricing environment in the US much more commercially appealing. Consequently, manufacturers often prioritize seeking MA in the US first, perpetuating access delays or resulting in complete lack of access in the EU.

### Challenges arising from increased usage of MEAs

MEAs are employed to manage evidentiary challenges in order to achieve market access for innovative therapies. Due to the challenges in assessing long-term efficacy and safety, outcomes-based MEAs are used extensively and are often the only route for CGT to gain market access in Europe (Ronco et al. 2021; Iglesias-López et al. 2023). Currently, all single-dose gene therapies available in Italy, the UK, France, Germany, and Spain are assigned to outcomes-based MEAs (Lee and Lee 2023). Unfortunately, outcomes-based MEA discussions add an additional delay (averaging 480 additional days) and increase the administrative burden of an already lengthy reimbursement negotiation process (Efthymiadou and Kanavos 2022). Furthermore, a major criticism of MEAs is their lack of transparency, which increases administrative burden, pricing inequality, and impacts patient access across the EU (Iunes et al. 2019; Russo et al. 2021; Greco et al. 2024). The details of the agreement, amount of discount, and sometimes even the existence of an MEA, are confidential (Russo et al. 2021; Thanimalai et al. 2021). Due to the lack of transparency, MEA negotiations may result in divergent contract terms in each country. Establishing separate terms with each national authority results in a significant duplication of work, which not only directly delays patient access to the negotiated product but also occupies resources from the evaluation of other drug products.

It has been argued that MEA confidentiality could enable governments with fewer healthcare resources to negotiate larger rebates. In reality, pricing confidentiality introduces a dramatic information asymmetry in favor of the manufacturer, which allows them to disregard external market influences, often disadvantaging countries with lower bargaining power. Such countries face challenges in negotiating favorable P&R agreements, ultimately leading to higher drug list prices in countries that have lower healthcare budgets (Morel et al. 2017; Young et al. 2017; Kostadinov et al. 2024). Moreover, despite the pretense of providing discounts and rebates, MEAs have been suggested to increase prices for both drugs with and without MEAs (Gamba et al. 2020; Carl and Vokinger 2021). When a manufacturer anticipates the use of an MEA, their proposed price is inflated to account for revenue lost through the discount (Gamba et al. 2020). It is therefore likely that CGT asking prices are inflated due to their heavy reliance on MEAs to achieve market access. There are multiple ways that the use of MEAs could also increase prices for drugs that do not normally rely on them to achieve reimbursement. First, pharmaceutical companies are likely to increase the asking price for any drug in anticipation of an MEA, regardless of if an MEA is utilized in the end (Gamba et al. 2020). Second, the increased administrative burden on both pharmaceutical companies and national authorities to maintain the MEA terms inevitably puts a financial burden on both parties, which increase overhead regulatory expenditures and may be reflected in drug list prices (Vreman et al. 2020). Third, a country using external reference pricing will overpay for a drug if they are referencing the list price in a country with a confidential MEA discount (Vogler et al. 2012; Kanavos et al. 2020; Palomo et al. 2023; Greco et al. 2024). With more innovative therapies entering the EU market, the short-term benefits of MEAs may be overcome by accumulating costs in multiple areas, and MEAs would increasingly burden the healthcare system.

## Addressed challenges, remaining gaps, and future directions

It is important to note that the arguments presented in this paper focus on the EU context, where member states share relatively high income levels, regulatory frameworks, and strong political and economic ties. The EU considers solidarity in healthcare a core value, as reflected in Article 35 of the Charter of Fundamental Rights (European Union Agency for Fundamental Rights 2007). Persistent systematic access inequalities between EU states risk undermining these principles, leading to avoidable healthcare inequities. Some structural features of the HTA framework may inadvertently perpetuate cross-border access disparities. While healthcare systems alone cannot resolve all access inequalities between European countries, redesigning certain HTA processes and increasing coordination between European countries could play a role in mitigating these disparities and upholding the EU’s commitment to healthcare solidarity.

Examining the drawbacks of HTA and P&R frameworks in a sector-specific way can overemphasise delays experienced by certain patient groups, while overlooking their role in promoting equitable access across the healthcare system. It is important to acknowledge the advantages and disadvantages of HTA and P&R frameworks for a more balanced approach to improving patient access. HTA and price-control mechanisms in Europe are important to ensure cost-effectiveness and sustainability in healthcare systems. While maintaining the benefits of the existing HTA-P&R frameworks, it is paramount to minimize patient harm by reducing avoidable access delays and cross-border inequalities. European countries are making progress towards optimizing HTA practices for expensive, innovative therapies, but there remains a gap in the operationalization of additional elements of value and developing standards for the use of real-world evidence and surrogate endpoints (Besley et al. 2023). Widening the scope of cost-benefit evaluations to capture additional elements of value may allow recognition of the value of innovative treatments beyond immediate clinical outcomes. Ideally, these changes would be implemented on an EU-wide scale, but countries with nationalized, public healthcare systems may be better equipped to consider non-clinical values, which have greater socioeconomic benefits but do not directly affect healthcare costs.

There are multiple initiatives to shorten the delays and enable increased access to new drugs in the EU. As mentioned previously, joint clinical assessments have the potential to greatly minimize discrepancies in time to access across the EU. We believe that this is a promising initiative towards increased speed and equal access to new therapies, especially expensive CGT, and look forward to broader implementation of joint clinical assessments for more drug classes in the future. Multiple EU member states have implemented regulatory and procedural mechanisms to mitigate access delays introduced by the HTA process. Some countries have specialized reimbursement policies for ATMPs. These pathways sometimes entirely forego HTA in exchange for immediate patient access. For example, in the Netherlands, ATMPs are eligible for full reimbursement immediately after EMA approval if they fall under a certain cost threshold (Rare Impact 2020). Other strategies preserve the full HTA framework while enabling earlier generation of cost-effectiveness evidence to support faster and more efficient evaluation and negotiation processes. In some countries, it is possible to apply for P&R before MA is granted, allowing HTA to be conducted concurrently with MA assessments (Jansen and Amesz 2020). This reduces the delay to patient access while maintaining the same duration and rigor of performance evaluation. Additionally, early benefit assessment is a strategy employed by the German HTA body to enable immediate patient access while also supporting long-term price regulation. The time to reimbursement is shorter in Germany than any other country in the EU, making it the most attractive market to launch in for many manufacturers (Danzon and Epstein 2012; European Federation of Pharmaceutical Industries and Associations 2023). Under the German AMNOG procedure, new products undergo an early benefit assessment in the first three months after MA (Gemeinsamer Bundesausschuss). During this time, the product is allowed on the market with full reimbursement while the HTA and pricing negotiations take place. This is only viable for healthcare systems with relatively larger budgets, as it requires the full reimbursement of a freely priced drug for the first few months of market access. It may also allow the collection of real-world evidence to inform concurrent HTA evaluations. One critical consideration with this strategy is that overspending on inefficacious drugs during this time may syphon healthcare budgets from other areas, harming patients by reducing the overall standard of care. To minimize this risk, we propose that health authorities could allow products onto the market with narrow indications before the HTA is complete. For example, a product could be reimbursed only for certain disease severities. Although price-control mechanisms inherently create commercial barriers, it is generally unsustainable for companies to launch CGTs exclusively in the US, and Europe remains a large and valuable market. We hope that by considering strategies such as concurrent HTA and early benefit assessment, countries in the EU can minimize disincentives in order to ensure timely patient access while maintaining rigorous cost-benefit evaluations.

ATMPs must be administered in specialized clinics, which have high infrastructural, administrative, and training costs. Different healthcare budgets, population sizes, and disease prevalence make it unrealistic for every health technology to be made available in every country. Under EU regulation, all patients are entitled to cross-border care (European Commission 2019). However, reimbursement of cross-border care is determined on a patient-by-patient basis for specialized and expensive treatments like CGT (European Commission 2019). Depending on the degree of insurance coverage, patients forced to seek cross-border treatment may have to pay for travel and healthcare expenses out-of-pocket. Greater coordination between national authorities could help to create a more harmonised and equitable union and prevent unfair competition on discounts for expensive therapies between member states, advancing the EU’s commitment to solidarity in healthcare. If it is unsustainable to provide access to certain treatments in one’s own country, national authorities could consider implementing policies that facilitate or reimburse cross-border healthcare. However, to make cross-border access functional in practice, cost barriers must still be addressed. One potential strategy, as recently arose from discussions at the ARRIGE 2025 Annual Meeting, involves collective purchasing mechanisms for high-cost therapies, where multiple EU states could negotiate shared pricing or pooled procurement agreements (ARRIGE 2025; Kaló et al. 2025). These approaches could reduce price disparities and increase bargaining power, facilitating more consistent access to advanced therapies for countries with fewer resources. 

Finally, we would like to acknowledge that optimizing HTA and P&R represents only one facet of the complex regulatory and legal issues surrounding fair access and pricing to CGT in Europe. Intellectual property rights (IPRs), such as patents and copyrights, trade secrets, regulatory data and market exclusivities, price controls, and competition/antitrust law, add crucial layers to the debate which we did not discuss. These may also have a significant impact on investment incentives for pharmaceutical innovation and collaboration, price limits, and regulations that ensure a healthy competition and innovation ecosystem. Well-designed industry policies and push and pull tools can act as economic incentives. For example, publicly funded grants, tax credits for R&D and facility investment, more flexible regulatory market exclusivities, and research grants could address economic risk in the rare disease areas with small patient numbers (Lee and Lee 2023). However, increasing investment incentives may also lead to tensions with affordability and access; such activities must be monitored, limited, enforced, and governed (e.g., via antitrust laws) (Kianzad and Minssen 2018).

Investment incentives should be combined with new regulatory approaches involving increased harmonization and a forward-looking and communicative regulatory framework that enables effective use of real-world data to resolve safety and efficacy uncertainties (Lee and Lee 2023). The development of innovation ecosystems and technology transfer frameworks in each country may play an important role in increasing CGT access. Market and policy innovations requirewell-coordinated action and dialogue among all stakeholders to ensure patients can benefit from new drugs while achieving overall health system sustainability (Phares et al. 2024). The recent EU pharmaceutical package developments, including new incentive models and regulatory proposals such as the Hospital Exemption pathway and regulatory sandboxes, will be especially significant in that regard.

## Conclusion

Assessing the economic impact of innovative, expensive therapies such as CGT is challenging due to uncertain long-term safety and efficacy. This complicates HTA and P&R discussions, leading to access delays or lack of reimbursement, and exacerbating access disparities between nations. Many health authorities have turned to MEAs to provide access to expensive drugs like gene therapies. While useful as an interim solution, they provide uncertain benefits and are difficult to assess due to their confidentiality, making them an unsustainable solution to provide access to expensive drugs in the long term. Health authorities should focus on increasing cross-border collaboration and developing HTA methods that are better able to assess disruptive health technologies.

## Data Availability

No datasets were generated or analysed during the current study.
